# Comparison of β-D-Glucan and Galactomannan in Serum for Detection of Invasive Aspergillosis: Retrospective Analysis with Focus on Early Diagnosis

**DOI:** 10.3390/jof6040253

**Published:** 2020-10-28

**Authors:** Karl Dichtl, Johannes Forster, Steffen Ormanns, Heidi Horns, Sebastian Suerbaum, Ulrich Seybold, Johannes Wagener

**Affiliations:** 1 Max von Pettenkofer-Institut, Medizinische Fakultät, LMU München, 80336 Munich, Germany; dichtl@mvp.lmu.de (K.D.); suerbaum@mvp.lmu.de (S.S.); 2 Institut für Hygiene und Mikrobiologie, Julius-Maximilians-Universität Würzburg, 97080 Würzburg, Germany; johannes.forster1@hygiene.uni-wuerzburg.de; 3 Pathologisches Institut, Medizinische Fakultät, LMU München, 80337 Munich, Germany; Steffen.Ormanns@med.uni-muenchen.de; 4 Klinik für Infektiologie und Tropenmedizin, München Klinik, 80804 Munich, Germany; heidi.horns@muenchen-klinik.de; 5 Sektion Klinische Infektiologie, Medizinische Klinik und Poliklinik IV, Klinikum der Universität, LMU Munich, 80336 Munich, Germany; Ulrich.Seybold@med.uni-muenchen.de; 6 Department of Clinical Microbiology, Trinity College Dublin, St James’s Hospital Campus, D08 RX0X Dublin, Ireland; 7 Nationales Referenzzentum für Invasive Pilzinfektionen (NRZMyk), 07745 Jena, Germany

**Keywords:** BDG, beta-D-glucan, GM, galactomannan, IA, invasive aspergillosis, biomarker, fungal antigens, serology

## Abstract

The early diagnosis of invasive aspergillosis (IA) relies mainly on computed tomography imaging and testing for fungal biomarkers such as galactomannan (GM). We compared an established ELISA for the detection of GM with a turbidimetric assay for detection of the panfungal biomarker β-D-glucan (BDG) for early diagnosis of IA. A total of 226 serum specimens from 47 proven and seven probable IA cases were analysed. Sensitivity was calculated for samples obtained closest to the day of IA-diagnosis (d0). Additional analyses were performed by including samples obtained during the presumed course of disease. Most IA cases involved the respiratory system (63%), and *Aspergillus fumigatus* was the most frequently isolated species (59%). For proven cases, sensitivity of BDG/GM analysis was 57%/40%. Including all samples dating from –6 to +1 weeks from d0 increased sensitivities to 74%/51%. Sensitivity of BDG testing was as high as or higher than GM testing for all subgroups and time intervals analysed. BDG testing was less specific (90–93%) than GM testing (99–100%). Combining BDG and GM testing resulted in sensitivity/specificity of 70%/91%. Often, BDG testing was positive before GM testing. Our study backs the use of BDG for diagnosis of suspected IA. We suggest combining BDG and GM to improve the overall sensitivity.

## 1. Introduction

Invasive aspergillosis (IA) is a life-threatening and underdiagnosed fungal infection that is caused by moulds of the genus *Aspergillus* [1]. The most important risk factor for IA is impaired host defence. Especially patients undergoing myeloablative therapies and hematopoietic stem cell transplantation (HSCT) are at highest risk [2,3]. In recent years, several other risk factors have been identified, such as systemic steroid therapy, intensive care unit admission, and influenza [4,5,6,7]. Despite availability of antifungal drugs, mortality of IA is extraordinarily high and ranges from 30 to 90% [7,8,9,10]. Besides underlying disease and immunosuppression, primary determinants for survival are early diagnosis and early antifungal therapy [11]. Clinical signs of IA are nonspecific, they may include cough, shortness of breath, and fever [12]. Diagnosis therefore relies on risk assessment combined with additional diagnostic evidence, such as characteristic signs in computed tomography (CT) scans, cultural growth *Aspergillus* spp., or positive *Aspergillus* biomarkers [2,3]. The use of nucleic acid testing for IA is still controversial. The 2019 update of the EORTC/MSG definitions of IA, which are intended for scientific purposes but not for guiding patient care, include PCR-positivity in the criteria for mycological evidence for probable IA [13]. Current clinical guidelines recommend to consider PCR only in conjunction with other diagnostic tools, particularly antigen testing [2,3]. To date, only two antigens are routinely used as biomarkers of systemic *Aspergillus* infections: 1) galactomannan (GM), a cell wall carbohydrate that is mostly specific for *Aspergillus* spp. GM testing is well-established and routinely performed in many diagnostic laboratories [11,14,15,16]. Current guidelines rate GM as being an accurate marker of IA and therefore recommend its measurement for diagnosis as well as screening purposes in high-risk populations [2,3]. 2) β-D-Glucan (BDG) is a major constituent of most fungal cell walls. It can be detected in blood of patients with invasive fungal infections such as invasive candidiasis, *Pneumocystis* pneumonia, and IA [17,18]. Several assays for BDG detection were developed and clinically approved in Japan in the early nineties. However, these tests were rarely used in other areas of the world. Only one test system, the Fungitell assay (Associates of Cape Cod, East Falmouth, MA), has been CE (Conformité Européenne) marked, approved by the United States Food and Drug Administration (FDA), and commercially available in the United States and Europe for many years. To date, there is a lack of large-scale studies that evaluate BDG testing in the setting of IA. Due to limited evidence, current guidelines state that the use of BDG should be restricted to specific purposes [2,3].

In the present study we compared the performance of the Fujifilm/Wako BDG assay (Fujifilm Wako Chemicals Europe GmbH, Neuss, Germany) and the Platelia *Aspergillus* GM ELISA (Bio-Rad Laboratories, Marnes-la-Coquette, France) in a large cohort of IA cases. Our results suggest that BDG testing is less specific but more sensitive and indicates IA earlier than GM testing.

## 2. Materials and Methods

This study was performed at the Max von Pettenkofer Institute for Hygiene and Medical Microbiology hosting the central microbiology laboratory for the University Hospital of Ludwig Maximilians University (LMU) Munich, a 2000 bed university medical centre in Munich, Germany. We retrospectively identified 47 and seven episodes of proven and probable IA according to the EORTC/MSG criteria (revision and update 2019), which occurred in the period of 2009–2018 (Table 1, Table 2 and Table 3) [13].

In one patient, we identified two episodes of probable IA with a 10-month interval and multiple negative GM tests in between. The day of sampling of the specimen that allowed for the diagnosis of proven and probable IA was defined as day of proven and probable diagnosis, respectively. Subsequently, this day will be referred to as day 0. Because of this, day 0 does not represent the date of the onset of infection but the time point of diagnosis.

Serum samples of 154 individuals were included as a control cohort consisting of two subgroups without mycological evidence for IA (Table 1). Seventy-six patients belong to the high-risk group for invasive fungal infections due to a history of myeloablative therapy and HSCT (≤ 7d before sampling). The remaining 78 individuals were outpatients with a suspicion of borreliosis (low-risk group for IA).

In total, 380 samples (stored at –20 °C) from IA cases and a control group were included in this study. For a more detailed analysis, we divided the sera of proven IA cases into three (overlapping) subsets (Table S1): The smallest subgroup (named “d 0”) consists of only one serum sample per case which was obtained closest to day 0 (54 samples with a maximum distance from day 0 of ±7 days). The second and the third subsets include 126 sera that were sampled in the period of ±7 days from day 0 (“±7 d”) and 183 sera dating from –6 weeks to +1 week from day 0 (“–6/+1 w”), respectively. The d 0 subgroup was used to calculate sensitivities of the tests. Analysing the ± 7d and the –6/+1 w subgroups, we aimed to evaluate whether the assays were able to establish a serologic diagnosis of IA at any time point during the course of infection. This seropositivity fraction will be referred to as “per case sensitivity”.

BDG measurements were conducted using the Wako BDG assay according to the manufacturers’ instructions (FUJIFILM Wako Chemicals Europe, Neuss, Germany). GM analysis was performed using the Platelia *Aspergillus* antigen ELISA (Bio-Rad Laboratories, Hercules, CA, USA). Optical density (OD) indices were rounded to one decimal place with a lower limit of 0.1. The respective cut-offs were 11 pg/ml for BDG and an OD index of 0.5 for GM. Statistical analysis was performed using Graphpad Prism 5 (GraphPad Software, La Jolla, CA, USA).

This retrospective study was reviewed and approved by the ethics committee of the university hospital of Munich (Ethikkommission der Medizinischen Fakultät der LMU München) and a waiver of informed consent was granted.

## 3. Results

### 3.1. Study Population

Forty-seven (87%) patients with proven and seven (13%) episodes of probable IA according to the revised definitions of the EORTC/MSG consensus group were included in this study [13]. A total of 226 serum samples of IA patients was available (mean and median of four samples per case; Table 1) acquired between –98 and day +51 from day 0. Most common focus of infection was the respiratory system (63%). Seventy-nine percent of proven IA cases were identified upon positive culture with *Aspergillus fumigatus* representing the dominant species. Details on the type of infection and patient characteristics are summarised in Table 2 and Table 3.

The control group consisted of 154 sera obtained from 78 outpatients with suspected borreliosis and from 76 patients at high risk of IA (hematopoietic stem cell transplantation ≤ 7days before sampling) who had no histologic or cultural evidence of IA (Table 3).

### 3.2. Sensitivity and Specificity of Tests

**Figure 1:**
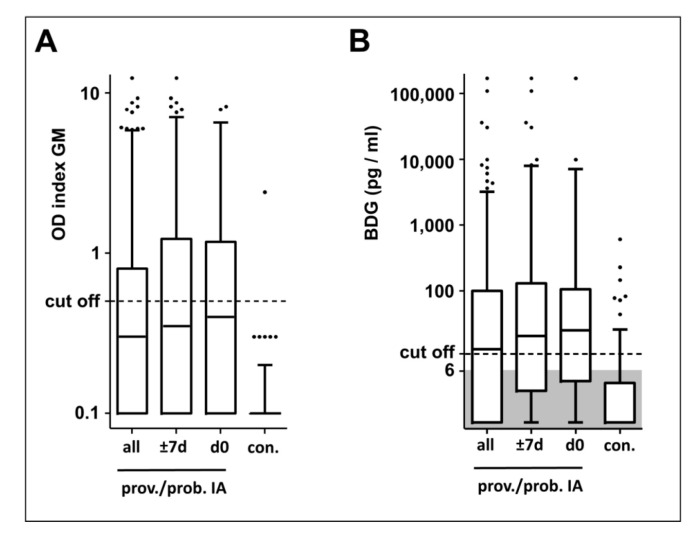
Distribution of GM and BDG measurement results. Three subsets of serum samples of proven/probable IA patients and of an *Aspergillus*-negative control group were analysed with the galactomannan (GM; (**A**)) EIA and the β-1,3-D-glucan (BDG; B) assay. The three subsets of sera of IA patients include (1) all available sera, (2) all sera obtained in the period of ± 7 days from the day of proven/probable diagnosis (= day 0), and (3) the single serum sampled closest to day 0 of each patient. The optical density indices (A) and the BDG concentrations (**B**) are depicted as box plots. Results below the limit of detection (6 pg/mL) were plotted not to scale in the shaded area. Whiskers mark 5 and 95 percentiles. Dotted lines indicate the applied cut-off levels for the individual tests (index of 0.5 for the GM ELISA and a concentration of 11 pg/mL BDG for the BDG assay). OD optical density; d days; prov. proven; prob. probable; con. controls.

Results of patients with proven/probable IA and controls are plotted in Figure 1. Notably, the median of GM measurements of all IA sample groups (d 0, ±7 d, all available samples) is below the EIA cut-off while the BDG median is above the respective cut-off. Focusing on the 54 sera sampled closest to day 0, sensitivities of GM and BDG are 48% and 56%. However, the seven included cases of probable IA are defined by GM seropositivity. Considering only cases of proven IA, the sensitivity of GM and BDG was 40% and 57%, Table 4). Upon suspicion of IA in high-risk patients, current guidelines recommend serial antigen testing in order to increase the sensitivity of serology [2,3]. Therefore, we included additional sera from the period of ±7 days from day 0 (Figure S1). The GM EIA and the BDG test yielded positive results in 47% and 68% of cases of proven IA, respectively (Table 4).

Importantly, day 0 does not represent the date of the onset of infection but the time point of diagnosis. Therefore, we extended the observation period and included all sera sampled between –6 to +1 weeks from day 0. BDG analysis identified considerably more episodes of proven IA than GM measurement in this subgroup (74% vs. 51%).

Next, the performance of the tests in different subpopulations with regards to focus of proven infection and underlying conditions was analysed. Notably, the GM EIA did not demonstrate superior sensitivity in any analysed subset (Table 4). We found a considerably higher sensitivity and per case sensitivity (herein defined as at least one seropositive sample in the indicated time period) for BDG testing in almost all subgroups. Both assays demonstrated a better performance in cases of non-respiratory IA (Table 4). Per case sensitivities of the GM EIA were notably higher in hematologic than in non-hematologic patients. In contrast, the differences between these subgroups were negligible when BDG analysis was applied. In consequence, superior sensitivity and per case sensitivity of BDG testing was most prominent in non-hematologic IA patients (sensitivities of 59% vs. 34% and ±7 d per case sensitivities of 69% vs. 41%).

In contrast, the GM EIA clearly outperforms the BDG assay in terms of specificity (99% vs. 90–93%, Table 4, Figure 1). In the control group of 78 patients with suspected borreliosis, 70 were negative in the BDG assay (90% specificity). Moreover, in the control group of 76 patients at high risk of IA, 71 were negative in the BDG assay (93% specificity). Only one out of 154 was positive (highly positive) for GM (99% specificity).

In a next step, the potential of a combination of both tests was investigated (Table 4). Defining seropositivity as both BDG- and GM-positivity, specificity increased to 100%, but sensitivity decreased dramatically (27% in the d0 sample group). However, if all sera in which at least one antigen was detected were classified positive, sensitivity increased to 70%, 81%, and 85% for the d 0, the ±7 d, and the –6/+1 w group. Comparable results were obtained upon exclusion of all sera sampled after d 0 (Table S2). The cohort of non-respiratory IA is particularly noteworthy: including all samples, 95% of cases were identified. Compared to the BDG-based measurement, there was no loss of specificity (91%).

**Figure 2:**
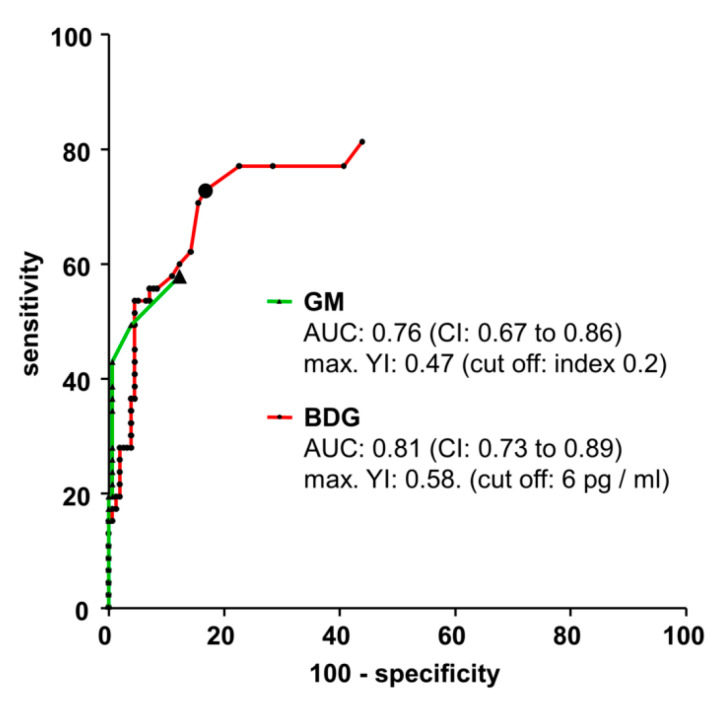
Receiver operating characteristic (ROC) curves. Measurement results of the samples of the control group and the single serum sampled closest to the day of proven diagnosis each IA patient were used to calculate ROC curves for the GM ELISA (triangles) and the BDG assay (circles). Data points with the maximum Youden’s index (YI) for the individual curves are labelled by large symbols. AUC area under the curve; CI confidence interval.

For each assay a receiver operating characteristic (ROC) curve was plotted using the measurement results of the d 0 samples group (proven IA) (Figure 2). The area under the curve was 0.76 for GM and 0.81 for BDG testing. The ROC analysis resulted in a maximum Youden’s index for a GM cut-off index of 0.2 (0.47) and for a BDG cut-off of 6 pg/ml (0.58). Applying the ROC analysis-based cut-off, the sensitivities of GM and BDG testing showed an increase from 40% to 60% and from 57% to 77% in the group of proven IA cases (plus nine cases each). However, the ROC analysis-based cut-offs decrease BDG specificity to 83% and GM specificity to 88%.

### 3.3. Time Point of Seropositivity

**Figure 3:**
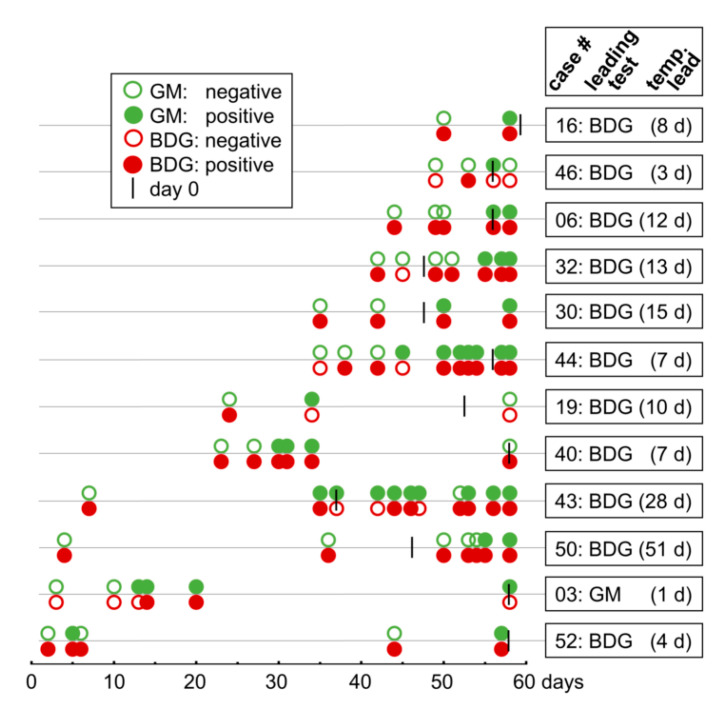
Comparison of seropositivity over the time course of infection. The time courses of twelve cases of proven/probable IA were plotted in which both tests were positive but seropositivity occurred at different time points (x-axis in days). Each case is displayed as separate line with green and red circles representing serum samples analysed with the galactomannan (GM) EIA and the β-1,3-D-glucan (BDG) assay, respectively. Empty and full circles indicate negative and positive measurement results, respectively. Black lines indicate the respective day 0 (day of proven/probable diagnosis of IA). Boxes at the right indicate the test which yielded positive results at the earlier time point and the temporal distance (in days) between the earliest dates of seropositivity of both assays, temp, temporal.

In 31 episodes of IA (57%) both tests yielded concordant results: in seven episodes, neither GM nor BDG was detected and in 24 episodes, both tests were positive at some point during the course of infection. Notably, in twelve of these 24 episodes, one test detected IA at an earlier time point. The time courses of seropositivity of these twelve cases are depicted in Figure 3. Seropositivity occurred significantly earlier with the BDG test than with the GM test (*p* < 0.01, Wilcoxon signed rank test). Remarkably, the BDG assay was the first test that detected IA in eleven of the twelve cases. In these eleven cases, the temporal lead ranged from 3–51 days (mean of thirteen and median of nine days). Up to five follow-up sera had to be analysed until the first positive result of the GM EIA (mean and median of two samples). Notably, in nine of the eleven cases the initial BDG test was positive, suggesting that BDG testing might have yielded positive results at even earlier time points. In contrast, the single case where the GM assay was the first positive test is characterised by a temporal lead of only one day. Nevertheless, all twelve vary with respect to the number and sampling time points of available sera. More frequent sampling might therefore still have reduced the time lag upon onset of GM seropositivity.

## 4. Discussion

IA is the most frequent invasive fungal disease in the high-risk population of neutropenic patients [19]. However, due to the rigid case definitions for research purposes studies investigating the diagnostic potential of BDG analysis are hampered by small case numbers [13]: We only identified twelve studies evaluating the test performance based on a cohort of more than twenty episodes of proven or probable IA according to the EORTC/MSG criteria [13,20,21,22,23,24,25,26,27,28,29,30,31]. Still, the vast majority of IA cases included in these studies was identified by GM seropositivity which may have biased comparison of different serological diagnostic tools. To date, our study comprehends the largest cohort of proven cases (*n* = 47) for comparison of the two serological biomarkers BDG and GM for diagnosis of IA.

Sensitivities or per case sensitivities of 48% to 79% have been reported for BDG testing in previous studies that included a considerable number of cases [20,21,22,23,24,25,26,27,28,29,30,31]. This is in a similar range as the sensitivity of 57% and per case sensitivity of 74% in the –6/+1 w subgroup in the present study. Importantly, for all studies investigating the performance of BDG for the diagnosis of IA, it has to be considered that BDG is a panfungal marker and seropositivity cannot be unequivocally attributed to *Aspergillus* spp. but might be caused by another, undetected fungal infection, such as *Pneumocystis* pneumonia or invasive candidiasis. Notably, none of these previous works investigated the Wako BDG assay, which is based on a different method (turbidimetric read-out) than the other available testing systems (colorimetric read-out). Our results suggest a higher specificity of BDG testing compared to most previous reports. This is most likely linked to the use of the Wako assay whose manufacturer’s defined cut-off is known to result in a higher specificity but also lower sensitivity compared to other BDG tests [20,21,22,23,24,25,26,27,28,29,30,31,32,33]. Nevertheless, a specificity of 92% still limits the use of the Wako BDG assay as a non-combined screening tool [2,3].

In contrast to the ground-breaking studies that established GM serology for the diagnosis of IA almost 20 years ago, the GM EIA demonstrated a surprisingly low sensitivity of only 40% in our cohort [34,35,36,37]. Importantly, these previous studies analysed GM performance in different and very focused patient cohorts. This might explain the overall much lower sensitivity observed in our study: (1) While the previous studies focused on cohorts with hematologic patients, our study population included individuals with a broad range of underlying conditions for IA. (2) It is very likely that several hematologic patients in our cohort received an antifungal chemoprophylaxis with posaconazole (treatment data for individual patients were not available due to medical data protection). Non-hematologic conditions and posaconazole prophylaxis are known to reduce GM sensitivity [38,39,40]. These considerations and our results are in good agreement with more recent findings (GM sensitivity of 49%) [26]. Notably, the performance of GM testing from bronchoalveolar lavage fluid is not affected by the above-mentioned factors but was proven to be a valuable combination partner for serum BDG analysis [3,41].

Non-hematologic patients with IA are a relevant and growing subgroup in the group of IA patients and nowadays account for approximately 50% of all IA episodes [4,6]. Interestingly, we did not observe a reduced sensitivity for the BDG test in this subgroup. Nevertheless, hematologic patients still represent the most important high-risk group for IA [2,3]. In this group, sensitivities of both assays in the samples closest to day 0 were only 53%. Expanding the observation period drastically increased the per case sensitivities (67% and 80% for GM and BDG analysis in the –6/+1 w subgroup, respectively). This underlines the importance of repetitive testing in high-risk patients.

At a first glance the results of our present work might suggest that IA could be diagnosed earlier by BDG detection than by GM detection. Indeed, BDG seropositivity had a temporal lead over GM seropositivity in almost all cases (eleven of twelve). However, this comes at the cost of a significantly lower specificity of the BDG test compared to the GM test (92% vs. 99%). We therefore assume that this temporal lead of the BDG test is a phenomenon primarily caused by the manufacturers’ defined cut-offs for the respective tests: while GM testing is more specific but less sensitive, BDG testing is more sensitive but less specific. The ROC curve for the BDG assay indicates that, at least in our cohort, altering the cut-off would not improve the test’s specificity to a level similar to that of the GM assay. Therefore, BDG testing remains particularly useful in patients suspicious for IA but not for screening purposes.

This study has certain limitations. As with most studies investigating IA, the total number of cases is still low, so the calculation of statistical significances for the different analyses had to be omitted. Furthermore, due to data protection regulations, we did not have access to all clinical data that would have been of interest for the interpretation of the results, e.g., information on antifungal prophylaxis or therapy.

Finally, our data demonstrate that combining both tests, i.e., by defining positive suspicion for IA upon seropositivity of one of the two assays, remarkably increases the sensitivity. At the same time, the combination of both tests has only a minor impact on the overall specificity compared to BDG testing alone. Due to the very high specificity of the GM EIA, combined testing is not inferior to BDG-based testing alone in terms of specificity. We therefore suggest that BDG testing and GM testing should be used in combination in patients suspicious for IA.

## Figures and Tables

**Table 1:** Sample characteristics.

	n	%
all serum samples	380	100
serum samples of IA cases	226	59
samples of proven cases	196	52
samples of probable cases	30	7
serum samples of control patients	154	41
suspected borreliosis	78	21
control following HSCT	76	20

Serum was the only sample type included in this study. n number of samples; HSCT hematopoietic stem cell transplantation.

**Table 2:** Characteristics of infection

	n	%
**EORTC/MSG category**		
proven IA	47	87
by culture	37	79
by histology only	9	19
probable IA*	7	13
**focus of IA**		
respiratory tract	34	63
intraabdominal	6	11
circulatory system	5	9
central nervous system	3	6
bones and joints	2	4
urogenital tract	2	4
orbita	1	2
skin	1	2
**isolated species**		
proven cause of infection		
*A. fumigatus*	32	59
*A. flavus*	3	6
*A. niger*	1	2
cultivation only from non-sterile body site		
*A. fumigatus*	7	13

Cases are categorized as “proven/probable IA (invasive aspergillosis)” following the revised definitions of the EORTC/MSG consensus group [13]. Cases of proven IA in which *Aspergillus* was cultivated from a sterile body site are listed in the subsection “proven cause of infection”. Cases in which the proven diagnosis relies on histological findings but in which *Aspergillus* was cultivated in a non-sterile body site are listed in the subsection “cultivation only from non-sterile body site”. * Two episodes of probable IA were diagnosed in the same patient; CNS, central nervous system; n, number of cases.

**Table 3:** Demographic characteristics and underlying conditions of IA patients

	n	%
**demographic characteristics**		
all cases of IA	54	100
female sex	21	39
age		
mean	56	
median	60	
**underlying conditions**		
hematologic malignancy	22	41
history of HSCT	13	24
solid organ transplantation	15	28
lung	8	15
heart	3	6
liver	3	6
kidney	1	2
intensive care treatment	9	17
solid organ malignancy	3	6
immunosuppressive therapy	2	4
predisposition unclear	2	4
cystic fibrosis (treated in ICU)	1	2

The category “immunosuppressive therapy” does not include patients with a history of transplantation who are listed separately. n number of cases; HSCT hematopoietic stem cell transplantation; ICU intensive care unit.

**Table 4:** Sensitivities and specificities.

	GM	BDG	GM ∧ BDG	GM ∨ BDG
**sensitivity**				
sera closest to day 0	40%	57%	27%	70%
hematologic disease	53%	53%	33%	73%
other underlying conditions	34%	59%	25%	69%
focus: respiratory system	33%	52%	22%	63%
focus: non-respiratory	50%	65%	35%	80%
**per case sensitivity**				
sera ± 7 days from day 0	47%	68%	34%	81%
hematologic disease	60%	67%	40%	87%
other underlying conditions	41%	69%	31%	78%
focus: respiratory system	41%	63%	30%	74%
focus: non-respiratory	55%	75%	40%	90%
sera – 6 to + 1 week from day 0	51%	74%	40%	85%
hematologic disease	67%	80%	53%	93%
other underlying conditions	44%	72%	34%	81%
focus: respiratory system	41%	70%	33%	78%
focus: non-respiratory	65%	80%	50%	95%
**specificity**				
all control sera	99%	92%	100%	91%
suspicion of borreliosis	99%	90%	100%	91%
control following BMT	100%	93%	100%	91%

Only cases of proven invasive aspergillosis (IA) were included in this analysis. Per case sensitivity is defined by at least one seropositive sample in the indicated time period. GM ∧ BDG, positive results with both assays; GM ∨ BDG, positive result of at least one test.

## Supplementary Materials

The following are available online at https://www.mdpi.com/2309-608X/6/4/253/s1, Figure S1: Results of antigen testing in the week before and after the day of proven or probable diagnosis; Table S1. Sample subgroups of IA cases analysed in this study; Table S2. Sensitivities calculated after exclusion of all sera that were sampled after day 0.
