# Supplementary material for: Comparison of β-D-Glucan and Galactomannan in Serum for Detection of Invasive Aspergillosis: Retrospective Analysis with Focus on Early Diagnosis
Source: J Fungi (Basel). 2020 Oct 28;6(4):253. doi: 10.3390/jof6040253 (PMC7711951; doi:10.3390/jof6040253)
Supplement: Supplementary file 1 [file jof-06-00253-s001.pdf]

Supplementary Figure 1

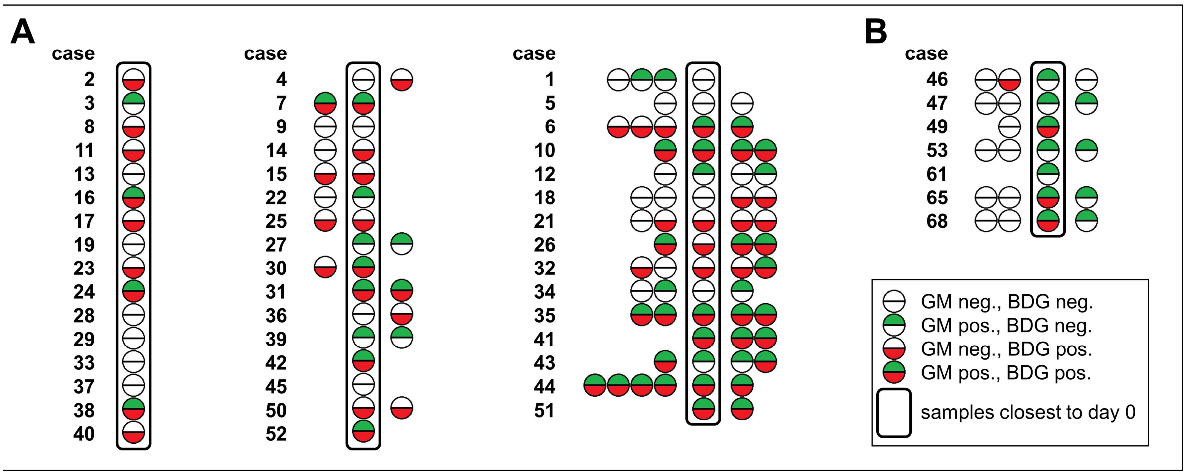

figure S1. Results of antigen testing in the week before and after the day of proven or probable diagnosis. 126 serum samples of 54 cases of proven (A) or probable (B) IA were analysed with the galactomannan (GM) ELISA and the β-1,3-D-glucan (BDG) assay. Each circle represents a serum sample (depicted in chronologic order). Sera sampled closest to the day of proven (A) or probable (B) diagnosis are framed. Green and red semicircles indicate positive test results for GM and BDG testing, respectively. Empty semicircles indicate negative test results. neg. negative; pos.

Table S1. Sample subgroups of IA cases analysed in this study. Each subgroup is also a subset of the next larger subgroup in the following line.

subgroup	distance from day 0	sera per case	sera in subgroup
d 0	-7 to +7 days	1	54
±7 d	-7 to +7 days	1 – 6	126
-6 / +1 w	-42 to + 7days	1 – 10	183
all sera	-98 to + 51 days	1 – 11	226

Table S2. Sensitivities calculated after exclusion of all sera that were sampled after day 0. All observation periods end at day 0. Only cases of proven invasive aspergillosis (IA) were included in this analysis. Per case sensitivity is defined by at least one seropositive sample in the indicated time period. Column "excluded cases" indicates the number of proven cases that was excluded because no sera were sampled in the analysed periods. GM ∨ BDG, positive result of at least one test.

	GM	BDG	GM ∨ BDG	excluded cases
**sensitivity**				
sera closest to day 0	41 %	54 %	69 %	8
**per case sensitivity**				
all sera in the period of day -7 to day 0	42 %	67 %	75 %	11
all sera in the period of week -6 to day 0	45 %	68 %	78 %	7
